# Anchoring Property of a Novel Hydrophilic Lipopolymer, HDAS-SHP, Post-Inserted in Preformed Liposomes

**DOI:** 10.3390/nano9091185

**Published:** 2019-08-21

**Authors:** Rosario Mare, Huining Da, Massimo Fresta, Donato Cosco, Vibhudutta Awasthi

**Affiliations:** 1 Department of Health Sciences, University “Magna Græcia”, Campus Universitario “S. Venuta”, Building of BioSciences, I-88100 Catanzaro, Italy; 2 Department of Pharmaceutical Sciences, College of Pharmacy, University of Oklahoma Health Sciences Center, 1110 North Stonewall Avenue, Oklahoma City, OK 73117, USA

**Keywords:** liposomes, polyethylene glycol, long-circulating, superhydrophilic polymer

## Abstract

Polyethylene glycol (PEG)-phospholipids in long-circulating liposomes cause non-specific immune reactions; mainly attributable to negatively-charged phosphoryl s at the interface of PEG and phospholipid. We investigated a novel lipopolymer, by which a superhydrophilic polymer (SHP) is conjugated to a non-phospholipid N^1^-(2-aminoethyl)-N^4^-hexadecyl-2-tetradecylsuccinamide (HDAS). The modification of preformed liposomes HDAS-SHP, HDAS-PEG_2000_, and DSPE-PEG_2000_ were performed by post-insertion techniques. The efficiency of post-insertion and desorption rates, from the liposome surface, were determined. HDAS-SHP micelles showed highly positive zeta potential (+28.4 mV); zeta potentials of DSPE-PEG_2000_ and HDAS-PEG_2000_ micelles were −34.4 mV, and −3.7 mV, respectively. Critical micelle concentration predicted amphiphilicity of HDAS-SHP (CMC 2.58 µM) as close to that of DSPE-PEG_2000_ (CMC 2.44 µM). Both HDAS-SHP and HDAS-PEG_2000_ post-inserted with comparable efficiency (79%, and 73%, respectively), but noticeably lower than DSPE-PEG_2000_ (90%). The desorption rate of HDAS-SHP was close to that of DSPE-PEG_2000_ (0.53%/h, and 0.45%/h, respectively); the desorption rate for HDAS-PEG_2000_ was slightly more at 0.67%/h. Compared to plain liposomes, both HDAS-SHP- and DSPE-PEG_2000_-liposomes showed significant leakage of encapsulated Na-fluorescein isothiocyanate (FITC) upon incubation with serum. At the same time, both modified liposomes were found to suppress serum levels of the complement proteins, Bb and C4d. We infer that HDAS-SHP is a viable alternative to commonly-used PEG-phospholipid derivatives for stealth purposes.

## 1. Introduction

**Figure 1:**
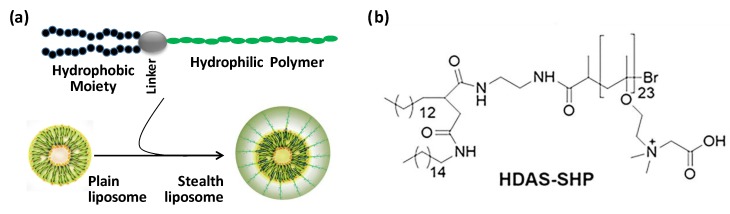
(**a**) Stealth lipopolymers are characterized by a hydrophobic anchor (phospholipid, etc.), a linker (phosphate or amide bond) and a hydrophilic polymer (PEG, etc.). (**b**) Structure of HDAS-SHP.

Liposomes have acquired wide acceptance as the nano-carriers of choice for pharmaceutical applications. Phospholipids are the main constituent of liposomes, but a composition, comprising of phospholipids and cholesterol, is more useful for drug delivery. The presence of cholesterol enhances the hydration of the lipid head group, stabilizes the membrane, and improves the retention of hydrophilic drugs [1,2]. The current success of liposomes as drug delivery systems could be largely credited to their surface-modification with polyethylene glycol (PEG)-conjugated amphiphilic lipopolymers, particularly phospholipid-based PEG-lipopolymers, such as PEG**_2000_**-distearoylphosphatidylethanolamine (DSPE) [3,4,5]. The presence of these amphiphiles on the liposome surface reduces opsonization and suppresses clearance by mononuclear phagocyte system (MPS), thereby prolonging the persistence of liposomes in circulation. Aptly called stealth liposomes, these preparations generally enable greater drug exposure, reduced toxicity, and less frequent administration. The stealth efficacy of PEG-phospholipids has been putatively explained by their ability to create a steric hindrance for the interaction with immuno-proteins, secondary to a highly hydrated surface of PEG molecules [6,7,8,9]. A typical phospholipid-based PEG-lipopolymer consists of a hydrophobic stearoyl or palmitoyl anchor, linked to a hydrophilic PEG chain (MW 2000–5000), via a phosphate ester linkage (Figure 1). 

Despite their widespread use, significant biological problems still exist with PEG-modified liposomes. These problems have been attributed to immune-related reactions of PEG moiety, as well as phospholipid anchors in the amphiphiles [10,11]. Immune reactions, associated with phospholipid anchors, are ascribed to the presence of a negatively-charged phosphoryl group in the phospholipids [12,13,14], and the nature of the acyl anchor [15]. These phospholipids can cause significant complement activation and associated pro-thrombocytopenic reactions [16,17]. For instance, liposomes containing negatively charged dimyristoylphosphatidylcholine (DMPG) can activate platelets [18], whereas, the net negative charge on the phosphate moiety of PEG-phospholipids has been reported to contribute in complement activation and anaphylotoxin production [12]. The liposome-mediated activation of the complement system can induce flushing and tightness of the chest in humans [19]. The use of PEG to hide the liposome surface from the immune system was initially thought to completely eliminate these issues of phospholipids. However, now we know that PEG is also not entirely innocuous. It can also induce complement-mediated hypersensitivity reactions [20,21], and its administration may generates anti-PEG IgM antibodies against the interface of PEG and phospholipid [22]. Circulating anti-PEG antibodies rapidly clear intravenously administered PEG-modified liposomes [23]. In fact, PEG that is conjugated to a phospholipid can induce a stronger immune reaction than PEG alone [12]. 

In recent years considerable effort has been directed towards the development of lipid amphiphiles, without the phospholipid component [10]. Towards this effort, we previously reported a synthetic anionic lipid devoid of phosphoryl moiety, *N*^1^-(2-aminoethyl)-*N*^4^-hexadecyl-2-tetradecylsuccinamide (HDAS), and followed it up by creating a lipopolymer HDAS-PEG for stealth modification of liposomes [16,17]. Further, we developed a super-hydrophilic polymer (SHP), poly [n-(carboxymethyl)-2-(isobutyryloxy)-*N*,*N*-dimethylethanammonium], as a replacement for PEG, and synthesized a novel stealth lipo-polymer HDAS-SHP; HDAS-SHP can match the efficacy of PEG-phospholipids in enhancing circulation t_1/2_ of liposomes [5]. However, its interaction with the liposome-surface has not been fully characterized yet. In this article, we investigated the anchoring property of HDAS-SHP in bilayers of preformed liposomes, and evaluated its ability to suppress complement-activation in comparison with DSPE-PEG_2000_, both in vitro and in vivo.

## 2. Materials and Methods 

The phospholipids were obtained from Avanti Polar (Alabaster, AL, USA). Cholesterol (CHOL), chloroform, methanol, ammonium thiocyanate, and N-phenyl-1-naphthylamine were obtained from VWR Scientific (West Chester, PA, USA) and Thermo Fisher Scientific Chemicals (Waltham, MA, USA). All other chemicals were purchased from either, VWR Scientific (West Chester, PA, USA) or Sigma-Aldrich (St. Louis, MO, USA), and used without further purification.

### 2.1. Synthesis of HDAS-Lipopolymers

HDAS, HDAS-PEG_2000_, and HDAS-SHP were synthesized in-house and characterized largely as described in our previous articles [5,17].

### 2.2. Critical Micellar Concentration (CMC)

The CMC of lipopolymers, namely HDAS-SHP, HDAS-PEG_2000_, and DSPE-PEG_2000_, was determined by PC1 photon-counting spectrofluorimeter analyses (ISS, Champaign, IL, USA), as described elsewhere [17,24]. Hydro-alcoholic solution (10% v/v ethanol in phosphate-buffered saline) of *N*-phenyl-1-naphthylamine (NPN, 1 µM) was used as a fluorescent probe. The spectrofluorimeter is equipped with a 300 W high-pressure xenon arc lamp (45 mW/nm brightness at 275 nm); analytical settings were λ_ex_ = 340 nm and λ_em_ = 350–550 nm. The maximum wavelength peak (λ_max_) for each sample was manually selected after the analyses to estimate CMC. Briefly, increasing amounts of lipopolymer solution were added to the NPN solution, and allowed to interact for 30 min at room temperature. Shifts in λ_max_ of the emission spectrum of NPN versus lipopolymer concentration were plotted, in order to find an inflection point indicative of CMC value. In all the experiments, the background spectra of buffer alone was subtracted from the corresponding sample spectra.

### 2.3. Liposome Preparation 

Liposomes were made by a method consisting of homogenization and extrusion. We used DSPC as the preferred phospholipid, because of its higher melting temperature (T_m_ = 55 °C), and results in enhanced stability of the resultant liposomes, compared with the other commonly used phospholipid DPPC. Briefly, DSPC-CHOL (7:3 M ratio) was dissolved in the chloroform-methanol mixture (3:1). The ratio, 70:30% has been reported to provide the most stable formulation [2]. After filtration, through a 0.2 µm nylon filter, the solvent was removed on an R-210 rotary evaporator (Buchi Corporation, New Castle, DE, USA) to obtain a thin film of lipid phase deposited inside a round bottom flask. The film was hydrated with milliQ^®^ water (~20 mg/mL) and passed 5 times through Emulsiflex C-3 Homogenizer equipped with an extruder (Avestin, Ottawa, ON, Canada). The extruder was presented with polycarbonate membranes (47 mm) of decreasing pore sizes (between 1 and 0.1 µm). The resultant preparation was centrifuged at 184,000× *g* for 30 min at 4 °C, using an Optima L-100 XP Ultracentrifuge (Beckman Coulter, Fullerton, CA, USA). The liposome pellet was suspended in water at phospholipid concentration of approximately 20 mg/L. 

### 2.4. Post-Insertion of Lipopolymers in Preformed Liposomes

Post-insertion reactions were performed by incubating preformed liposomes with micellar suspensions of HDAS-SHP, HDAS-PEG_2000_, and DSPE-PEG_2000_ (10 mole percent with respect to phospholipid concentration), as previously described [25]. The incubation temperature was set at 55 °C, a value close to the transition temperature T_m_ of DSPC; the reaction was allowed to proceed for an hour under slow and continuous stirring. Afterwards, the temperature was allowed to gradually equilibrate to room temperature, which favored the integration of the post-inserted molecules within the liposome bilayers. The resultant post-inserted liposomes were separated from un-incorporated lipopolymer by centrifugation at 137,000× *g* and 4 °C for 1 h in Beckman Optima L-100 XP ultracentrifuge. The liposome pellet was suspended in water and re-centrifuged to eliminate any residual free or micellar lipopolymer. After two wash-cycles, the pellet was re-suspended in water to phospholipid concentration of approximately 20 mg/mL. A portion of original liposome without post-insertion was kept for use as control liposomes. Particle size analyses were performed at the start of post-insertion (t_0_) and after post-insertion (t_1_). 

### 2.5. Phospholipid Concentration

The phospholipid concentration in liposomes was estimated by the Stewart colorimetric assay [26]. This method employs an aqueous reagent of ammonium ferrothiocyanate, prepared with ferric chloride hexahydrate (27.03 g/L) and ammonium thiocyanate (30.4 g/L). For the assay, liposomes (20 μL) were dried under nitrogen gas and the residue was dissolved in 2 mL chloroform. The color was developed by adding 2 mL of ammonium ferrothiocyanate solution, and by vigorously vortexing the two-phase mixture for 1 min. The mixture was then centrifuged (8500× *g*) to separate the organic layer for spectrophotometry at λ = 488 nm.

### 2.6. Dynamic Light Scattering

Liposome preparations and micellar lipopolymers were characterized by photon correlation spectroscopy (PCS), using a dynamic light-scattering Zeta PALS instrument (Brookhaven Instruments Corporation, Holtsville, NY, USA). This instrument has a 4.5 mW laser diode operating at 670 nm as light source, both for size and surface charge analyses; back-scattered photons were detected at 90° angle. The real and imaginary refractive indices were set at 1.59 and 0.0, respectively. The medium refractive index (1.330), medium viscosity (1.0 mPa s), and dielectric constant (80.4) were set before the measurements. Liposome samples were diluted 1:50 for dynamic light-scattering.

### 2.7. Anchoring Property of the Lipopolymers

The tendency for post-inserted lipopolymers to leave the liposome surface was evaluated by measuring the amount of lipopolymer released in dispersion medium under sink conditions. Briefly, the post-inserted liposomes were taken in Spectra/Por Float-A-Lyzer G2 dialysis devices (Spectrum Labs, Rancho Dominguez, CA, USA), equipped with 20 kDa cellulose membranes. The dialysis devices were suspended in milliQ^®^ water under gentle stirring at 37 °C. The dialysis chamber was sampled at pre-determined intervals, and the samples were assayed for the presence of lipopolymers by the I_2_-complexation method described below. The anchoring property of lipopolymers was deduced as a difference between the initial (zero time) concentration of lipopolymer and the concentration determined at various other times.

The lipopolymer concentration in liposomes was determined by using a variant of the I_2_-colorimetric assay, as described by Francois and De Neve in 1985 [27]. Briefly, liposome samples were treated with 20% v/v of two aqueous solutions- BaCl_2_ (5% w/v) in 1N HCl and a solution of KI (2% w/v) and I_2_ (1.27% w/v) in water. The I_2_-polymer complex was quantified at λ = 535 nm in a Synergy2^TM^ multiwall plate reader (Bioteck Instruments, Winooski, VT, USA). A sample of plain liposomes (without lipopolymer post-insertion) was used as a control. Post-insertion efficiency was calculated as a fraction of lipopolymer, initially added to the preformed liposomes.

### 2.8. Stability of Surface-Modified Liposomes in Serum

The complement attack on liposomes, modified with HDAS-SHP, was studied in vitro. Plain-liposomes, HDAS-SHP-liposomes, and DSPE-PEG_2000_-liposomes, containing Na-fluorescein isothiocyanate (FITC) were prepared by extrusion method and post-insertion of stealth amphiphiles, following a method described previously [17] and detailed in Appendix A. Aliquots of liposome preparations were incubated with normal human serum at 37 °C (1:5 v/v). To control the variation in immune reactivity of sera from one lot to another, we will use pooled sera type AB from clotted blood of male donors. (Sigma-Aldrich, St. Louis, MO, USA). Exogenous C-reactive protein (CRP, 10 µg) was replenished in select incubations. Identical incubations were also performed with CRP-depleted human serum (Fitzgerald Industries International, Acton, MA, USA). Liposomes, incubated without serum, served as the technical controls, and serum incubated without liposomes was taken as a background in these experiments. After 1 h of incubation, 500 µL of stopping solution (10 mM EDTA, 25 mg/ml BSA, and 0.05% Tween-20) was added to each reaction tube. Approximately 350 µL of the mixture was centrifuged in Galaxy 20R Centrifuge (VWR International, Radnor, PA, USA) to remove any liposome particles (17,000× g for 10 min at 4 °C). The supernatant was diluted with phosphate-buffered saline in ratios of 1:1, 1:2, and 1:4, and the dilutions were measured in triplicate at excitation λ = 440 nm/emission λ = 528 nm by microplate reader (BioTek, Synergy2). The total FITC, associated with liposome preparations, was estimated by mixing liposomes with equal volume of 2% of C_12_E_10_ detergent (Sigma-Aldrich) to dissolve the lipid and measuring fluorescence. 

### 2.9. Complement Activation by HDAS-SHP- and DSPE-PEG_2000_-Liposomes

The activation of complement by plain-liposomes, HDAS-SHP-liposomes, and DSPE-PEG_2000_-Liposomes was studied by incubating the preparations with normal human serum (1:5 *v*/*v*), as described above for 1 h at 37 °C. The mixtures were diluted with saline and centrifuged in Galaxy 20R Centrifuge (VWR International) to remove any liposome particles (17,000× g for 30 min at 4 °C). The supernatants were diluted 1:30 (Bb), 1:40 (SC5b), and 1:70 (C4d) and assayed for SC5b, C4d, and Bb using MicroVue human complement enzyme immune assay kits (Quidel, San Diego, CA, USA). The manufacturer’s recommended method was followed.

### 2.10. Complement Activation by HDAS-SHP- and DSPE-PEG_2000_-Liposomes in Mice

All animal experiments were conducted according to a protocol approved by the Institutional Animal Care and Use Committee of the University of Oklahoma Health Sciences Center. BALB/c mice (n = 10/group, 20–25 g) were recruited for this study. Three liposome preparations (plain liposomes, HDAS-SHP-liposomes, and DSPE-PEG_2000_-liposomes; Supplemental Material) and two controls (lipopolysaccharide (LPS, 250 ng), as a positive control and saline as negative control) were injected in the tail vein of mice under 2% isoflurane anesthesia. The volume of injection was 0.125 mL and lipid dose was maintained at 3.0 mg. After 1 h of injection, blood was collected by cardiac puncture and centrifuged to separate plasma. The concentrations of effector complement proteins C3a and C5a were determined in plasma samples by using mouse-specific enzyme-linked immune assay kits obtained from LifeSpan Biosciences (Seattle, WA, USA), and MyBioSource.com (San Diego, CA, USA), respectively. 

### 2.11. Data Analysis

The data were statistically analyzed by the univariate analysis of variance using Prism 8 software for Windows (GraphPad, La Jolla, CA, USA). All average values were given ± standard deviation (SD). Statistical comparisons between the two groups were performed using a two-tailed Student’s *t*-Test. The acceptable probability for significance was *p* < 0.05. For in vivo data, if the experimental positive and negative controls failed to give expected assay results, the entire set of data, to which that sample belonged, were excluded from the final calculations. 

## 3. Results

HDAS-SHP is a novel non-PEG and non-phospholipid alternative to PEG-phospholipids for surface modification of liposomes. In this work, we investigated the interaction of HDAS-SHP with preformed liposomes to define its anchoring ability on liposome surface.

### 3.1. CMC of HDAS-Lipopolymers and Characteristics of Resultant Micelles

**Figure 2:**
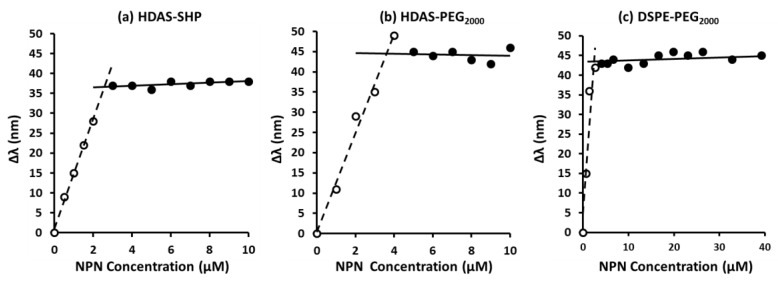
Critical micelle concentration (CMC) of (**a**) HDAS-SHP, (**b**) HDAS-PEG_2000_, and (**c**) DSPE-PEG_2000_. Hydro-alcoholic solution of N-Phenyl-1-naphthylamine (1 µM) was used as a fluorescent probe.

CMC is a concentration above which amphiphilic molecules exist as supramolecular aggregates or micelles. We determined the CMC values of HDAS-PEG_2000_ and HDAS-SHP by fluorometry; a widely used amphiphile DSPE-PEG_2000_ was used as a standard for comparison (Figure 2). HDAS-SHP presented the CMC value of 2.58 µM, which was close to the CMC value of 2.44 µM for DSPE-PEG_2000_. The CMC value for HDAS-PEG_2000_ was slightly higher at 3.61 µM. It is noteworthy that the *k_ASS_* values (slope of the dashed line, Figure 2) was also higher for HDAS-SHP (*k_ASS_* = 13.8) than that for HDAS-PEG_2000_ (*k_ASS_* = 12.2). These data suggest that, as compared to HDAS-PEG_2000_, HDAS-SHP exhibits a greater tendency to spontaneously form small and stable supramolecular micellar aggregates. 

We further investigated the micelle-forming characteristics of HDAS-lipopolymers and DSPE-PEG_2000_ by photon correlation spectro-fluorimetry at concentrations higher than their CMC. Dispersions of HDAS-amphiphilic derivatives in aqueous medium at 2 mg/mL provided micellar colloid with mean diameter less than 100 nm. Whereas, HDAS-lipopolymers formed significantly bigger micelles than DSPE-PEG_2000_ micelles, micelles composed of HDAS-SHP were significantly smaller than those obtained from HDAS-PEG_2000_; the polydispersity indices for all dispersions were narrow, regardless of the type of hydrophilic polymer present (PEG or SHP; Table 1). The type of hydrophilic portion attached to HDAS affected the surface charge (ζ-potential) of the resultant micelles. As expected, because of the presence of phosphatidyl groups, DSPE-PEG_2000_ micelles showed the highest negative ζ-potential value (−34.4 mV). On the other hand, HDAS-SHP micelles showed a ζ-potential towards positive value (+28.4 mV); HDAS-PEG_2000_ had an intermediate ζ-potential of −3.7 mV (Table 1).

### 3.2. Post-Insertion of HDAS-Lipopolymers and Their Retention in Liposome Bilayer

We evaluated the ability of HDAS-lipopolymers to modify surface of preformed liposomes by post-insertion technique. The preformed liposomes had a mean diameter of 120 nm with a narrow size distribution and ζ-potential of −12.8 mV (Table 2); the phospholipid concentration of preformed liposomes was 20.22 µM. Immediately after micelles were added to the preformed liposomes (time t_0_), there was an apparent increase in particle size for all derivatives considered (t_0_ size = 161–181 nm, Table 2), but more so in the case of DSPE-PEG_2000_ (*p* < 0.05 versus all others). After post-insertion was complete (time t_1_ = 1 h), the particle size of all preparations stabilized fairly close to each other (t_1_ size = 132–144 nm). The corresponding change in ζ-potential for various preparations was indicative of surface modification; the ζ-potential values post-insertion shifted towards values, which were close to those previously reported for corresponding micellar suspensions in Table 1.

**Figure 3:**
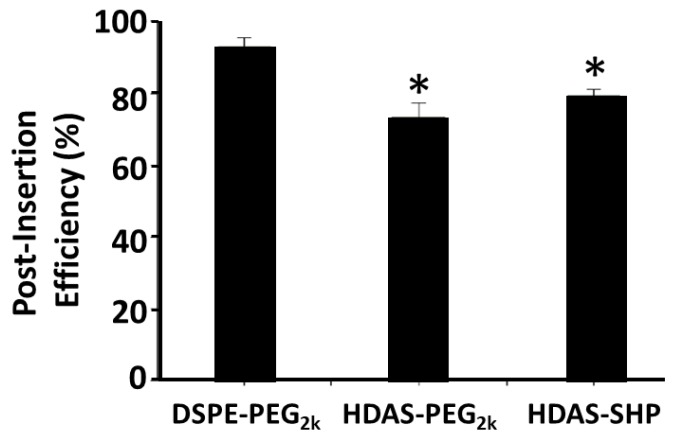
Post-insertion efficiency of various lipopolymers in preformed DSPC-CHOL liposomes (7:3 M ratio). Post insertion was performed for over 1 h incubation as described in the main text. The results are average of five different analyses ± sd (* *p* < 0.05 versus DSPE-PEG_2000_).

In order to determine the efficiency of post-insertion, we employed an assay based on complexation of molecular iodine by polymeric structure. This assay also confirmed the successful post-insertion reactions with both HDAS derivatives. HDAS-SHP purchased the higher post-insertion value, showing more than 79% incorporation in the liposome surface (Figure 3); HDAS-PEG_2000_ showed slightly lower insertion efficiency of 73%. In comparison, the post-insertion efficiency for DSPE-PEG_2000_ exceeded 90%.

**Figure 4:**
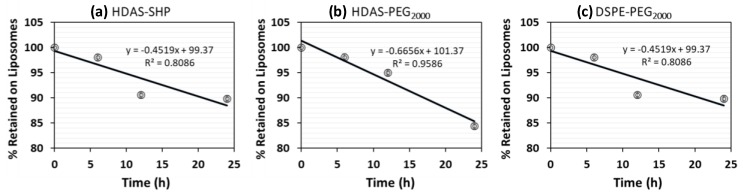
Desorption of (**a**) HDAS-SHP, (**b**) HDAS-PEG_2000_, and (**c**) DSPE-PEG_2000_ from liposome surface after post-insertion.

Next, we determined the fraction of each amphiphile released from liposomes, with respect to time, by allowing the liposomes to experience thermodynamic sink condition for up to 24 h at 37 °C. As shown in Figure 4, desorption of HDAS-lipopolymers and DSPE-PEG_2000_ approximated linear relationship with respect to time. After 24 h, approximately 10% and 15% of DSPE-PEG_2000_ and HDAS-lipopolymers were released in the medium, respectively (Table 3). In particular, HDAS-SHP (*k_DESORP_* = −0.53%/h) showed a profile of desorption close to the DSPE-PEG_2000_ (*k_DESORP_* = −0.45%/h). HDAS-PEG_2000_ was desorbed from the liposome surface at a relatively higher rate (*k_DESORP_* = −0.67%/h).

### 3.3. Liposome Stability in Serum and Effect of Liposomes on Complement Pathway

**Figure 5:**
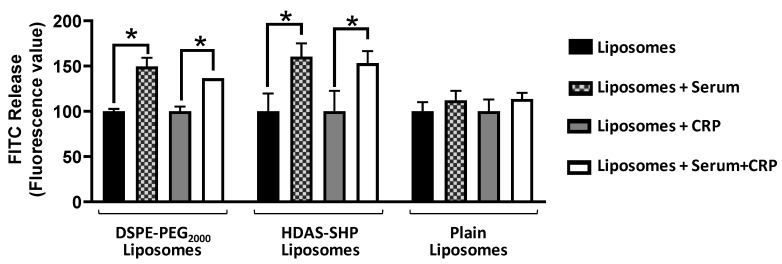
Effect of serum on fluorescein isothiocyanate (FITC) leakage from liposomes. Liposomes modified with HDAS-SHP and DSPE-PEG_2000_ were incubated with normal human serum with exogenous CRP. Unmodified liposomes (Plain) served as a control preparation. The data are representative of three separate experiments, each performed in triplicate (* *p* < 0.05).

**Figure 6:**
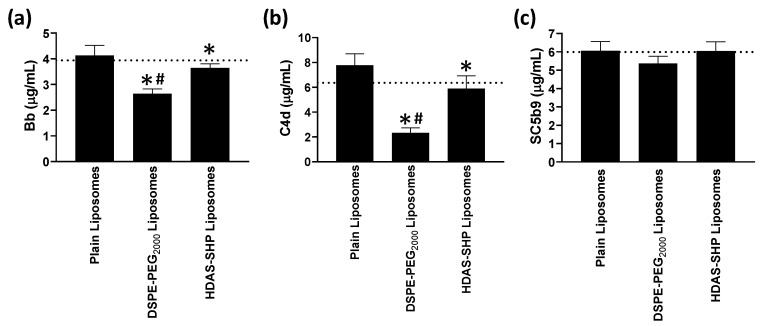
Effect of liposomes on serum complement proteins (**a**) Bb, (**b**) C4d, and (**c**) SC5b9. Liposomes modified with HDAS-SHP and DSPE-PEG_2000_ were incubated with normal human serum. Unmodified liposomes (Plain) served as a control preparation. The data are representative of three separate experiments, each performed at least in quadruplicate (* *p* < 0.05 versus Plain liposomes and ^#^ *p* < 0.05 versus HDAS-SHP liposomes).

Opsonizing proteins present in blood have a tendency to destabilize liposomes, resulting in leakage of encapsulated material. Therefore, we studied the effect of normal human serum on leakage of encapsulated FITC over a period of 1 h. The characteristics of FITC-liposomes are given in Supplementary Material. As shown in Figure 5, incubation of DSPE-PEG_2000_-liposomes and HDAS-SHP-liposomes with serum resulted in FITC leakage; this leakage was significantly higher than that observed with plain liposomes. The addition of exogenous CRP had no additional effect on FITC leakage. The non-specific opsonization of liposomes is mostly driven by complement proteins. Levels of C4d (classical marker), Bb (alternate marker), and SC5b9 (S protein-bound terminal complex) in the supernatant were estimated by the enzyme immunoassay (Figure 6). We found a significant suppression of complement proteins Bb and C4d in surface-modified liposomes, as compared to the plain liposomes. However, DSPE-PEG_2000_-liposomes also suppressed Bb and C4d significantly lower than the normal serum levels of these complement proteins; HDAS-SHP-liposomes were innocuous in this respect. There was no effect of any of the liposome preparations on the levels of SC5b9 (Figure 6c).

**Figure 7:**
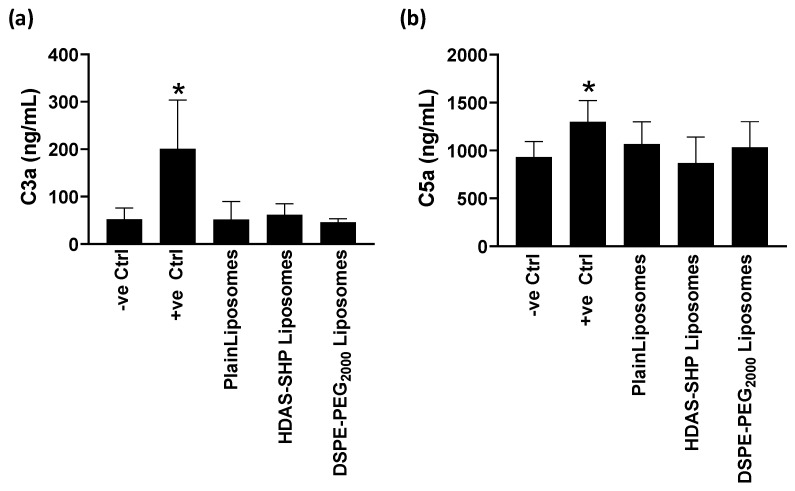
Effect of liposome injections on complement proteins (**a**) C3a and (**b**) C5a. Liposomes and control preparations were intravenously injected in mice and concentrations of C3a and C5a was estimated in plasma samples. The data are average of 4–7 samples per group (* *p* < 0.05 versus negative control).

Finally, we studied the activation of complement pathway by injecting plain liposomes, DSPE-PEG_2000_-liposomes, and HDAS-SHP-liposomes in mice and measuring C3a and C5a complement proteins in plasma (Figure 7). LPS and saline were injected as positive and negative controls, respectively. We found that none of the liposome preparations activated C3 and C5 complement proteins.

## 4. Discussion

HDAS was first developed as a replacement for anionic phospholipids in compositions of liposome-encapsulated hemoglobin; it helped increase encapsulation of hemoglobin and showed no tendency to activate platelets [16]. Later, we reported a PEG-linked conjugate of HDAS for enhancing circulation half-life of liposomes [17]. Compared to DSPE-PEG_2000_, HDAS-PEG_2000_ was found to have significantly reduced liposome-induced complement activation [17], plausibly because of a weaker anionic surface charge imparted by HDAS-PEG, as compared to DSPE-PEG_2000_, which contains a highly anionic phosphatidyl group (Table 1). More recently, we reported a conjugate of HDAS with SHP for the replacement of PEG-phospholipids [5]. The objective of this research was to investigate the anchoring characteristics of HDAS-SHP in preformed liposomes. 

As the chemical structure of SHP in Fig 1b indicates, it is a zwitterionic polymer, composed of multiple repeats of quaternary ammonium and carboxylate groups. Because of this, zwitterionicity SHP is highly hydrophilic and provides a sharp hydrophilic-hydrophobic interface [28,29]. The quaternary NH_4_^+^ exhibits a permanent positive charge in a wide range of pH values, whereas the carboxylate ion shows a negative charge or no charge, depending on the pH [30]. This is distinct from PEG, which has no permanent charge. Zeta potential values of micellar HDAS-SHP versus DSPE-PEG_2000_ clearly reflect this charge-associated distinction between the two lipopolymers (Table 1). As compared to the highly negative ζ-potential of DSPE-PEG_2000_, HDAS-SHP exhibits a positive ζ-potential. The presence of zwitterionic characteristics also ensures that SHP is strongly hydrated through electrostatic interactions. Hydration of PEG chains, on the other hand, is dependent on hydrogen-bonding. Therefore, SHP exhibits superior hydrophilicity, as compared to PEG, which is expected to increase its stealth properties [31,32]. Unlike SHP, PEG structure also contains hydrophobic domains, which results in a diffuse hydrophilic-hydrophobic margin and, in turn, can affect the stability of liposomes. The hydrophobic domains of PEG can interact with the underlying phospholipid bilayer and thwart the hydration of membrane phospholipid head-groups; poor hydration affects drug loading, destabilization of liposomes and difficulty in freeze-drying cycles [19,20,21,22].

Stealth lipopolymers for surface modification of liposomes are conventionally incorporated as a part of the lipid phase in the first stage of liposome making. However, this technique modifies both, external, as well internal surfaces of liposomes. The internal modification does not contribute to the stealth property of the liposomes; instead, it results in a reduced encapsulation-worthy space inside the liposomes, because the PEG brush or mushroom structures in the internal space can exclude the drugs and biomolecules from getting encapsulated [33]. In addition to undesirably restricting the internal aqueous space, the internal PEG-phospholipid is also amenable to acid/base-catalyzed hydrolytic degradation in pH gradient liposomes. The resultant hydrolysate can potentially compromise active loading and the retention of drugs inside such liposomes [34]. Equally noteworthy, is the issue of economics- expensive, but essentially ineffective internal PEG-lipid, which does not contribute to the conceived intention of enhanced circulation persistence, is wasteful. This wastage is significantly increased in the case of multi-lamellar liposomes, and disproportionately impacts internal space of smaller liposomes [35]. Realizing these problems, Uster et al. introduced the concept of post-insertion, as a technique where preformed liposomes were modified only at their external surface [36]. This technique has been successfully reported for many liposome-based drug and biologic delivery systems, and has now become central to liposome development in our lab [24,34,35,37,38]. In post-insertion technique, it is important to present stealth lipopolymers to the preformed liposomes in monomeric form. The micellar form, which exists at concentrations above CMC, does not favor efficient incorporation of stealth lipopolymers in lipid bilayers. 

CMC not only indicates the tendency of amphiphilic molecules to form small and stable supramolecular aggregates as micelles, but it also predicts the size and shape, as well as the stability of the micelles. The CMC of an amphiphile is affected by the type of hydrophilic polymer, as well as the lipophilic anchor. This is indicated by significant differences in CMCs of HDAS-SHP, HDAS-PEG_2000_, and DSPE-PEG_2000_. Whereas HDAS-SHP and HDAS-PEG_2000_ present the same lipid anchor and different hydrophilic moiety, DSPE-PEG_2000_ and HDAS-PEG_2000_ differ only in the type of lipid anchor. Despite presenting the same PEG hydrophile, DSPE-PEG_2000_ showed significantly lower CMC than HDAS-PEG_2000_. These observations and the calculated *k_ASS_* value suggested that HDAS-SHP has a greater tendency to form small and stable supra-molecular micellar aggregates, compared to HDAS-PEG_2000_. However, micelles, composed of HDAS-lipopolymers, were significantly bigger than DSPE-PEG_2000_ micelles, regardless of whether PEG or SHP was conjugated to HDAS. The dimensional differences between HDAS-SHP and DSPE-PEG_2000_ could be explained on the basis of structural differences, both, in hydrophilic polymer (PEG versus SHP), as well as in the anchoring moiety (DSPE versus HDAS). At the same time, a comparison between HDAS-PEG_2000_ and DSPE-PEG_2000_ showed that HDAS-PEG_2000_ formed micelles of much bigger hydrodynamic diameter, which suggested that the lipophilic components of amphiphiles influence the packing arrangement in the micellar form. It could be hypothesized that, as compared to HDAS-lipopolymers, DSPE-PEG_2000_ affords thermodynamically more stable micelles, which predicates a comparatively lower tendency to incorporate during post-insertion.

The tendency of a stealth lipopolymer to post-insert and anchor within the lipid bilayer of liposomes is driven by the above physicochemical characteristics of lipopolymers, and the same was found to be true for HDAS-SHP, DSPE-PEG_2000_, and HDAS-PEG_2000_. Dynamic light scattering data in Table 2 suggest that at the start of incubation, micelles of all three amphiphiles showed a tendency to adsorb on the liposome surface, which resulted in an apparent increase in the t_0_ liposome size from 120 nm to 161–181 nm. DSPE-PEG_2000_ showed higher t_0_ size, possibly due to the lower stability of its micelles, as compared to micelles of HDAS-PEG_2000_ and HDAS-SHP. Lower polydispersity indices for HDAS-PEG_2000_ and HDAS-SHP also support this conjecture. After the completion of post-insertion, the particle size stabilized to its final levels (t_1_ size), however liposomes post-inserted with DSPE-PEG_2000_ remained slightly larger than liposomes post-inserted with HDAS-SHP or HDAS-PEG_2000_. Thus, even though DSPE-PEG_2000_ showed higher post-insertion efficiency (90% for DSPE-PEG_2000_ versus 79% for HDAS-SHP), HDAS-modified liposomes showed more compact lipid packing and perhaps a better spatial disposition of hydrophilic portions. 

The prolonged circulation of surface-modified liposomes in vivo is dependent on how long the stealth-imparting amphiphile persists on the liposome surface. A recent study in our lab suggested that the desorption of PEG-lipopolymers from post-inserted liposomes is a thermodynamic process, which is time- and dilution-dependent [17]. Parr et al. has previously investigated the retention of PEG-coating on the liposome bilayer, and found that the removal of PEG, in vivo, is dependent on the nature of the lipid portion, and the characteristic of the linkage between the lipid and PEG [39]. Our in vitro experiments, simulating the in vivo sink conditions and body temperature of 37 °C, showed that the rate of desorption (*k_DESORP_*) for HDAS-PEG_2000_ was noticeably higher, as compared with the rate of desorption for DSPE-PEG_2000_ and HDAS-SHP (Figure 3; Table 3). Desorption for all three amphiphiles appeared to follow a linear profile, with correlation values of 0.89, 0.81, and 0.96 for HDAS-SHP, DSPE-PEG_2000_, and HDAS-PEG_2000_, respectively. The similarity of desorption profile of HDAS-SHP and DSPE-PEG_2000_ suggest that HDAS-SHP has the ability to effectively enhance the circulation half-life of modified liposomes. A recent pharmacokinetic study in rats also showed that, liposomes modified with HDAS-SHP are comparable to the liposomes, modified with DSPE-PEG_2000_ in this respect [5]. 

The clearance of liposomes from circulation is primarily influenced by the complement system [40,41,42]. A complement-dependent reduction in circulating platelets, immediately following the infusion of liposomes, has also been reported [43]. Studies in various animal models have revealed that the presence of anionic phospholipids in liposome compositions is the main cause of complement activation [12,42,44]. Since, HDAS-SHP is devoid of anionic phosphoryl group, we hypothesized that liposomes, that have been surface-modified with HDAS-SHP, will not activate complement proteins. To investigate this, we first examined both, classical and alternative complement activation pathways, in vitro, by determining the complement proteins C4d (classical marker), Bb (alternate marker) and SC5b (S protein-bound terminal complex). The classical complement pathway is initiated by an antibody or by C1-complex formation, whereas the alternate complement pathway is mediated by C3b directly on pathogen surfaces [45]. HDAS-SHP-liposomes reduced these markers in pooled serum, as compared to the plain liposomes, but not lower than the basal levels. Further, in the in vivo experiments, we did not find the effector complement proteins, C3 and C5, were activated. Overall, these results suggested that post-inserted HDAS-SHP prevents reactions which have been classified as complement-activation-related pseudoallergy or CARPA [7,20].

## 5. Conclusions

PEG is non-ionic and highly soluble, in both aqueous and organic media, biocompatible, with low immunogenicity, and good excretion kinetics. Its highly hydrophilic nature provides steric hindrance against protein adsorption and recognition by macrophages. Therefore, PEG-phospholipids are the most commonly employed materials for creating stealth liposomes. However, realizing a long-circulating liposome formulation, without the use of PEG-phospholipids, presents a thrilling challenge for pharmaceutical technology. This work demonstrates the utility of a novel non-PEG-non-phospholipid HDAS-SHP, as a replacement for PEG-phospholipids. HDAS-SHP formed small and stable micelles, at very low concentration, and these micelles intercalated in preformed liposomes to produce surface-modified vesicles, with a simple post-insertion process. Just like phospholipid-based stealth lipopolymer DSPE-PEG_2000_, HDAS-SHP was found to be well-integrated and retained by the liposomes. We conclude that HDAS-SHP represents an innovative tool for stealth purposes and serves as a viable substitute to PEG-phospholipids. 

As a new pharmaceutical excipient, HDAS-SHP is likely to face considerable regulatory hurdle. Several liposomal formulations have been approved for clinical use, but the use of a novel lipopolymer for the coating of vesicles, may represent a sticking point in clinical translation, especially because of PEG legacy and general lack of regulatory standards. To assert immune-neutrality, as well as stealth efficacy of HDAS-SHP relative to DSPE-PEG_2000_, it is essential to conduct a well-controlled comparative study in an appropriate animal model. Accordingly, future studies will also entail comprehensive toxicology of HDAS-SHP. For surface modification of liposomes, HDAS-SHP will directly compete with PEG lipopolymers at an economic level as well. It is important to control the synthesis of HDAS-SHP to ensure its purity and reproducibility of physicochemical characteristics, which brings the question of scalability. As reported in a previous article [5], the synthesis of SHP polymers, based on atom transfer radical polymerization, could easily be scaled up; the chemicals required for SHP synthesis are available in bulk at reasonable costs. The synthesis of the HDAS component could also be easily scaled-up, because its major precursor (tetradecenyl succinic anhydride) is available in large quantities in the petroleum industry [16]. Since the manufacturing process should exhibit consistent product quality, future scale-up and testing of HDAS-SHP must consider the regulatory requirements of chemistry, manufacturing and controls, and good manufacturing practices. 

## Figures and Tables

**Table 1:** Particle size of micelles of different lipopolymers. Results are presented as mean ± SD (* *p* < 0.05 versus DSPE-PEG_2000_ and ^#^ *p* < 0.05 versus HDAS-PEG_2000_).

Micelles	Size (nm)	Polydispersity	ζ-Potential (mV)
DSPE-PEG_2000_	29 ± 5	0.069 ± 0.02	−34.4 ± 2.3
HDAS-PEG_2000_	71 ± 2 *	0.073 ± 0.03	−3.7 ± 0.8 *
HDAS-SHP	53 ± 4 *^,#^	0.063 ± 0.01	+28.4 ± 0.5 *^,#^

**Table 2:** Size characteristics of liposomes before (t_0_) and after (t_1_) post-insertion. Results are presented as mean ± SD (* *p* < 0.05 versus Preformed liposomes and ^#^ *p* < 0.05 versus DSPE-PEG_2000_).

Liposomes	Size (nm)		Polydispersity		ζ-Potential (mV)	
	t_0_	t_1_	t_0_	t_1_	t_0_	t_1_
Plain	120 ± 2	-	0.092 ± 0.02	-	−12.8 ± 1.2	-
DSPE-PEG_2000_	181 ± 2 *	144 ± 1 *	0.081 ± 0.03	0.038 ± 0.02	−39.6 ± 3.1	−47.3 ± 3.7
HDAS-PEG_2000_	169 ± 2 *^,#^	132 ± 2 *^,#^	0.042 ± 0.02	0.038 ± 0.01	−9.1 ± 0.71	−8.7 ± 0.9
HDAS-SHP	161 ± 3 *^,#^	135 ± 2 *^,#^	0.076 ± 0.03	0.038 ± 0.01	+39.1 ± 3.1	+33.5 ± 2.6

**Table 3:** Desorption of lipopolymers (%) from DSPC:CHOL liposomes. Incubation was performed at 37 °C under sink conditions. The results are average of three different analyses ± SD.

Samples	Time (h)				*k_DISORP_*
	0	6	12	24	(%/h)
DSPE-PEG_2000_	100	98.04 ± 0.4	90.60 ± 0.7	89.84 ± 1.1	−0.45
HDAS-PEG_2000_	100	98.11 ± 0.3	94.99 ± 0.5	84.39 ± 0.9	−0.67
HDAS-SHP	100	92.79 ± 0.5	93.26 ± 0.3	86.24 ± 0.9	−0.53

## Appendix A

### Preparation of Fluorescein isothiocyanate-Loaded Liposomes

FITC Liposomes was made by extrusion method. Lipid phase 1,2-dihexadecanoyl-snglycero-3-phosphocholine (DPPC), 1,2-ditetradecanoyl-sn-glycero-3-phospho-(1-rac-glycerol) (sodium salt) (DMPG), cholesterol (CHO), and vitamin E in 45:10:44.8:0.2 mol% was dissolved in chloroform:methanol (2:1 v/v) system. After filtration through a 0.2 µm nylon filter, the solvent was removed on an R-210 rotary evaporator (Buchi Corporation, New Castle, DE, USA) to obtain a thin film of lipid phase in a round bottom flask. The film was hydrated with sterile milliQ^®^ water and the suspension was lyophilized overnight (Triad Lyophilizer; Labconco, Kansas City, MO, USA) to create pro-liposomes. The dried mixture was again hydrated with sterile 15 mL of 0.5 mg/mL FITC (Sigma-Aldrich, St. Louis, MO, USA) in cell culture grade water. The lipid suspension was sequentially extruded through polycarbonate membranes of pore sizes 1, 0.6, 0.4, and 0.2 µm in an extruder (Lipex Biomembranes Inc., Vancouver, BC, Canada). The resultant preparation was centrifuged at 184,000× *g* for 30 min at 4 °C by using an Optima L-100 XP Ultracentrifuge (Beckman Coulter, Fullerton, CA, USA). The supernatant was removed, the pellet was re-suspended in phosphate-buffered saline (PBS), and the centrifugation cycle was repeated two more times to completely eliminate extra-vesicular FTIC. The final liposome pellet was suspended in PBS at phospholipid concentration of approximately 20 mg/L (~27 mM with respect to DPPC).

### Post-Insertion of HDAS-SHP and DSPE-PEG_2000_

The insertion of lipopolymers in FITC liposomes was performed by essentially the method described in the main text. Briefly, 0.01 mmol (equivalent to approximately 7% of the total lipid in the liposomes) of lipopolymer was dissolved in 50 mL of water and filtered through 0.2 μm. The solution was gradually added to a diluted suspension of preformed liposomes using a programmable syringe pump (Chemyx Inc., Satfford, TX, USA). The mixture was gently stirred at 37 °C in inert (N_2_) atmosphere during the addition process. The preparation was washed free of any unincorporated lipopolymer by centrifugation at 184,000× *g* for 45 min at 5 °C (Optima L-100 XP ultracentrifuge, Beckman Coulter, Fullerton, CA, USA). The final pellet of surface-modified liposomes was resuspended in PBS (pH 7.4) and stored at 4 °C until further use.

To estimate the concentration of encapsulated FITC, an aliquot of liposome preparation was diluted 5 times with water and added with equal volume of 2% of C_12_E_10_ detergent (Sigma-Aldrich) to dissolve the lipid. The fluorescence of the resultant solution was measured in triplicate at excitation λ = 440 nm/emission λ = 528 nm by Synergy 2 microplate reader (BioTek, Winooski, VT, USA) and the concentration of FITC in the samples was determined against a standard curve from a [FITC] versus Fluorescence plot.

**Table A1:** Characteristics of FITC-liposomes.

Samples	Size, nm	Polydispersity	ζ-Potential, mV	[FITC], µg/mL
Plain Liposomes	162 ± 1	0.05 ± 0.01	−55.7 ± 2.7	1.41 ± 0.19
DSPE-PEG_2000_ Liposomes	172 ± 2	0.081 ± 0.03	−68.0 ± 1.1	0.81 ± 0.06
HDAS-SHP Liposomes	174 ± 1	0.050 ± 0.02	−59.9 ± 3.1	0.91 ± 0.12
